# THE ROLE OF CAREGIVERS DURING DISASTERS

**DOI:** 10.1093/geroni/igac059.629

**Published:** 2022-12-20

**Authors:** Tamar Wyte-Lake, Leah Haverhals

## Abstract

The COVID-19 pandemic laid bare the critical role caregivers play in supporting the older adult population, and how easily care structures can fall apart under the stress of a disaster event. As our population rapidly ages, it is imperative to better understand how to support caregivers to ensure relationships between caregivers and older adults remain robust and guarantee everyone’s safety. Therefore, this symposium focuses on the roles of formal and informal caregivers during disasters, primarily the COVID-19 pandemic. Dr. Richard Chunga will present findings from a survey of homecare aides, exploring drivers of high turnover rates and describing how employers can improve job satisfaction. Dr. Lindsay Peterson will describe barriers and facilitators to disaster preparedness among caregivers, using interviews with caregivers from diverse backgrounds in Florida. Ms. Jessica McLaughlin (PhDc) will share experiences of informal female caregivers, derived from interviews across the United States (US). Chelsea Manheim (LCSW) will describe adaptations to care provision for rural Medical Foster Home (MFH) Veterans from interviews conducted with MFH care providers from across the US. Dr. Carrie Wendel-Hummell will share data around the strengths and challenges of the self-directed care model for home-based long-term care, drawing on interviews with consumers, caregivers, workers, and providers in Kansas. As a whole, these presenters will provide insights into experiences of caregivers as they navigated challenges associated with the COVID-19 pandemic and generate forward thinking on how to inform future disaster response. Sponsoring SIGs: Paid Caregiving, Family Caregiving, Assisted Living, Disaster and Older Adults

